# Dynamic Perceptual Changes in Audiovisual Simultaneity

**DOI:** 10.1371/journal.pone.0001253

**Published:** 2007-12-05

**Authors:** Ryota Kanai, Bhavin R. Sheth, Frans A. J. Verstraten, Shinsuke Shimojo

**Affiliations:** 1 Division of Biology, California Institute of Technology, Pasadena, California, United States of America; 2 Department of Electrical and Computer Engineering, University of Houston, Houston, Texas, United States of America; 3 Center for NeuroEngineering and Cognitive Sciences, University of Houston, Houston, Texas, United States of America; 4 Department of Experimental Psychology, Helmholtz Institute, Universiteit Utrecht, Utrecht, The Netherlands; University of Sydney, Australia

## Abstract

**Background:**

The timing at which sensory input reaches the level of conscious perception is an intriguing question still awaiting an answer. It is often assumed that both visual and auditory percepts have a modality specific processing delay and their difference determines perceptual temporal offset.

**Methodology/Principal Findings:**

Here, we show that the perception of audiovisual simultaneity can change flexibly and fluctuates over a short period of time while subjects observe a constant stimulus. We investigated the mechanisms underlying the spontaneous alternations in this audiovisual illusion and found that attention plays a crucial role. When attention was distracted from the stimulus, the perceptual transitions disappeared. When attention was directed to a visual event, the perceived timing of an auditory event was attracted towards that event.

**Conclusions/Significance:**

This multistable display illustrates how flexible perceived timing can be, and at the same time offers a paradigm to dissociate perceptual from stimulus-driven factors in crossmodal feature binding. Our findings suggest that the perception of crossmodal synchrony depends on perceptual binding of audiovisual stimuli as a common event.

## Introduction

Perception of crossmodal simultaneity is important for our perceptual system, as it indicates which information should be integrated across different sensory modalities [1]. Determining the temporal relationship between auditory and visual events, however, poses a challenge for our brain. The temporal relationship of the neuronal responses directly available does not correspond to the physical relationship. This is because the conduction times, both physical and neural, are different for visual and auditory stimuli. Moreover, in a natural environment, sensory stimuli of multiple sources can occur in close temporal proximity, imposing a correspondence problem in the time domain. Nevertheless, the brain has a remarkable ability to produce fairly good estimates of the actual temporal relationship across different modalities [2]: the brain can compensate for the signal conduction times dependent on the distance from the source audiovisual event [3]–[6], [but see 7], and can constantly calibrate the point of AV synchrony as shown by adaptation to artificial temporal delays [8]–[11].

**Figure 1:**
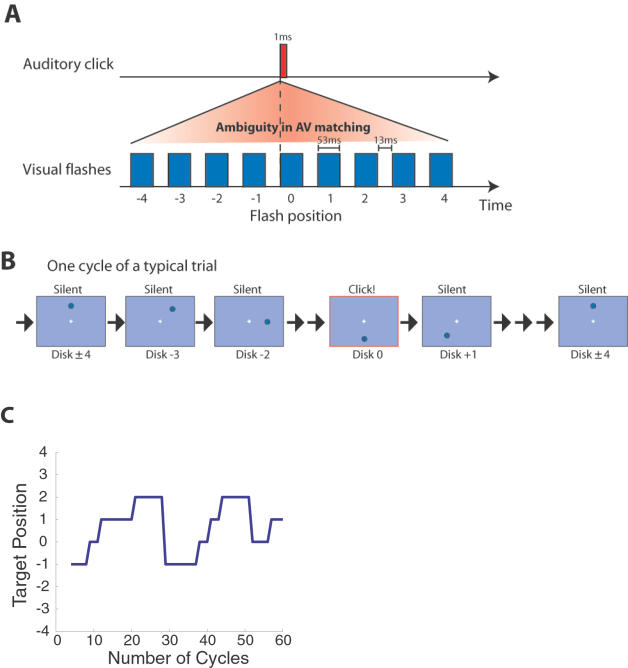
Multistability in AV temporal matching. A. Multiple visual flashes are presented in close temporal proximity to produce ambiguity in AV temporal matching. Many cycles were repeated continuously. B. A typical trial was illustrated. The visual flashes were presented at one of eight locations equidistant from fixation, producing the percept of a moving disk. One cycle lasted 534 ms. On a single trial, 60 identical cycles were repeated. C. The report of a representative trial is plotted as a function of stimulus cycles.

In the present study, we investigate how our perceptual system determines temporal correspondences when confronted with ambiguity. We deliberately introduced ambiguity by presenting multiple visual targets for a single auditory click (Figure 1A). The visual targets were disks flashed sequentially at one of eight locations, producing the percept of a disk revolving around fixation. The auditory click was presented at the same point in every cycle (534 ms), as the disk came to a particular location (Figure 1B). This stimulus was essentially identical to the complication clocks in classical psychological studies in which the perceived timing of a discrete auditory or tactile event was compared with respect to the position of a continuously moving visual stimulus [12]–[15]. However, in our stimuli, the visual events were discrete as opposed to continuous, and observations were made continuously, even after the first perceptual judgment on AV synchrony was made.

Although classical studies claim that sensations of simultaneity between different sensory modalities are less clear compared to sensations of simultaneity within the same modality [16], [17], more recent studies have demonstrated the ability to bind auditory clicks to visual events [1], [18]–[20]. In our stimuli too, observers reported that it was easy to identify a perceptually synchronous disk. These perceptually synchronous disks were seen as brighter with a sharper on and offset. Importantly, the disk that is seen as perceptually synchronous does not remain constant across cycles. Typically, observers report that the position at which simultaneity is perceived changes every 5 to10 seconds (i.e., 10–20 cycles).

The existence of multistability in the perception of this stimulus illustrates that the perception of AV synchrony is not fixed to a single point, but can dynamically change. Here, we use this phenomenon to examine the potential impact of attention on AV synchrony. We find an effect of attentional attraction, where perceived AV synchrony is attracted towards a visually attended event, regardless of its actual timing relative to the auditory stimulus. This suggest that the perception of AV synchrony is not determined simply by the perceptual latency for each modality, but is contingent upon the perceptual binding of auditory and visual stimuli as originating from a common event.

## Results

### Multistability in perceived AV synchrony

To characterize the basic transition pattern, we measured responses from ten observers while they continuously indicated the perceptually synchronous disk by holding down a corresponding key. On each trial, 60 consecutive cycles (∼32 s) were repeated, and each observer completed 32 trials.

**Figure 2:**
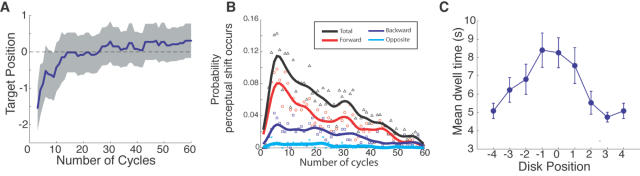
The basic characteristics of perceptual transitions in AV synchrony. A. The mean AV synchrony relative to the veridical position (n = 10). Positive values indicate forward shifts of perceived audiovisual synchrony. The gray zone represents one standard error of the mean. The data are plotted from the third cycle onwards as there was no response in earlier cycles due to response latency. B. Probability that a transition occurs in the forward (step size: +1, +2, or +3), backward (step size: −1, −2, or −3) or to the 180° opposite position (±4), which is directionally uncategorizable either as forward or backward, as a function of cycle number is shown. The black line is the probability sum of all transitions. The smooth curves are obtained by convolving the point (event) data with a Gaussian kernel (σ = 2 cycles). C. The mean dwell time is plotted as a function of disk position. The error bars indicate one s.e.m. (n = 10).

The results show that the initial perceived location of AV synchrony was biased towards a location in the disk sequence that occurred earlier than the physically synchronous location (Figure 2A). This is consistent with a number of studies showing that an auditory event is generally perceived earlier than a simultaneous visual event [4], [ 22]–[25], [but see 26], [27].

However, the position of perceived AV synchrony did change over time: As the stimulus cycles repeated, the perceived location of AV synchrony started shifting to other positions. The grand mean across all the observers and trials revealed a general tendency for the perceived AV synchrony to drift forward from the initial position, which is earlier than the position of physical synchrony. The dominance of the forward shift can be seen in Figure 2B: transitions occurred more frequently along the motion direction, especially for the early cycles of each trial.

The mean duration of each percept before the next transition is plotted as a function of the temporal position relative to the synchronous disk in Figure 2C. Not surprisingly, the percept dwelled longer at the near-veridical positions (over 8 s at disk positions −1 and 0), and the stability decreased for temporally more distant positions.

**Figure 3:**
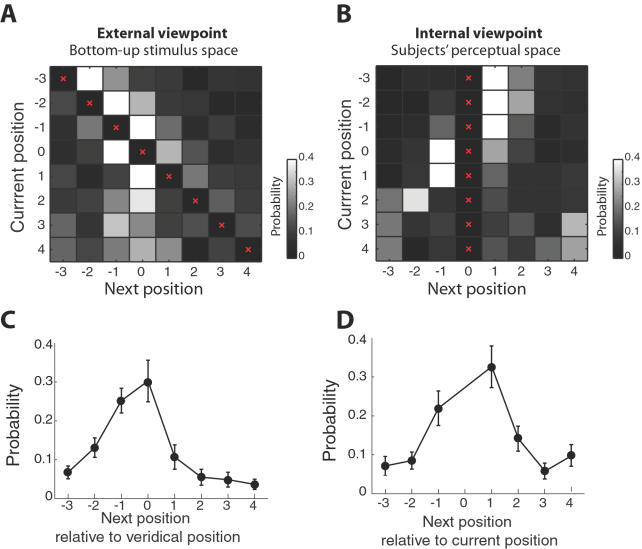
Characteristics of the group transition pattern. A. Transition probabilities are shown for all possible transition combinations. The diagonal elements signifying no perceptual shifts (red crosses) are not shown. B. The same data are represented relative to current position emphasize the directionality of the shifts. C. Transition probability marginalized across all current positions is plotted as a function of the absolute disk position. D. Transition probability marginalized across all current positions is plotted as a function of the relative disk position.

The analyses above suggest that some systematic trends are present in the perceptual switches. To fully characterize the transition pattern, we constructed a transition probability matrix from the data (Figure 3A). This representation of the data helps us to identify the dependency of the next position of perceived AV synchrony on the previous position. As can be seen in the probability distribution marginalized over current positions (Figure 3C), transitions were made most frequently to the near-veridical positions.

Realigning the matrix with respect to the current state (Figure 3B), it can be seen that the most frequent transitions were typically one-step forward from the current position (Figure 3D). This directionality is due to the bias in the forward transitions during the early cycles (see Figure 2A and Figure 2B). These two trends, that is, transitions towards the near-veridical positions, and forward transitions with respect to the current position signify, respectively, constraints on the flexibility of perception by bottom-up sensory signals and the contribution of current perceptual or attentional states.

### Sensory adaptation

What is the driving mechanism underlying the forward transition? The first possibility that may occur to one's mind is that the initial judgment is inaccurate because the task is too difficult and the sensory signals are too noisy, and repeated observations made the judgments more and more accurate over time. This is possible, but unlikely, because it cannot explain the initial bias and the systematic drift towards the more veridical range: that is, the observed shift was more systematic than just from a less accurate to a more accurate judgment.

**Figure 4:**
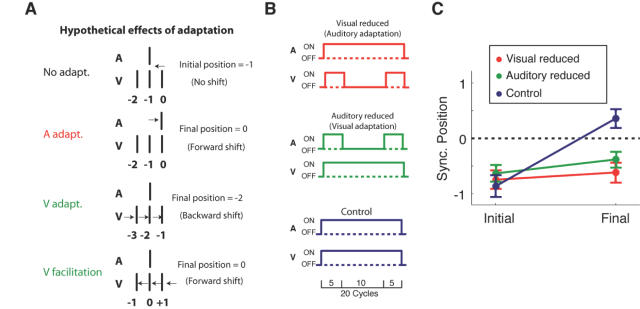
The experiment with a physical disruption of stimulus continuity. A. Hypothetical effects of adaptation are illustrated. Without adaptation, the auditory click is temporally aligned with the visual disk just prior to the veridically synchronous disk (top row). If audiotry adaptation would increase the processing latency for the auditory click, the position of the perceptually synchronous disk would shift forward (second row). Likewise, if adaptation to visual stimuli would increase the latency for vision, the position of the perceptually synchronous disk would shift backward (third row). If continuous presentation of visual disks would result in a shortening of processing latency, the position of the perceptually synchronous disk would shift forward. B. Three experimental conditions are schematically illustrated. In the flash off condition (top), the visual stimulus was turned off during the middle cycles and returned to the screen during the last five cycles. In the sound off condition (middle), the click sound was turned off during the middle cycles. In the control condition (botttom), there was no disruption during the middle cycles. C. The mean synchronous positions for the initial and the final cycles are plotted for each condition. The error bars indicate one s.e.m. (n = 6).

A more plausible mechanism is sensory adaptation. Inspection of Figure 2A shows that most forward transitions occurred in the initially 20 cycles (∼10 s) and then leveled off. The gradual leveling off is consistent with the general concept of adaptation. Possible effects of unimodal adaptation to either the visual or the auditory stimuli are illustrated in Figure 4A. If adaptation to the auditory click systematically delays the perceived timing of the auditory click, forward shifts would be observed by presenting the auditory click alone without the visual stimuli (Figure 4A, second row). Delays in processing the visual flash due to visual adaptation, however, would result in backward transitions (Figure 4A, third row)–an effect opposite to what was observed in the first experiment. Thus, a simple form of visual adaptation does not seem to account for the forward shift. Though unlikely, the hypothetical facilitation of visual processing speed by continuous exposures to the visual flashes could also result in a forward shift (Figure 4A, bottom row).

To examine these possibilities, we presented trials of 20 stimulus-cycles in which either the visual or auditory component was omitted for the middle 10 trials (Figure 4B). Compared to control trials in which both components were present throughout, these experimental trials had reduced levels of either visual or auditory adaptation leading into the final five stimulus cycles. The observer (n = 8) reported the position of AV synchrony only for the initial and the final cycle. The auditory adaptation hypothesis predicts that even in the reduced visual adaptation condition, continuous presentation of the auditory click during the middle cycles should produce the forward shift. On the other hand, if exposure to visual flashes is important for forward transitions, visual stimulation in the reduced auditory adaptation condition should still produce a forward shift.

The results are shown in Figure 4C. As expected, a clear forward transition was observed in the control condition (shift amount, 1.22±0.24 disk positions; paired t-test, t(7) = 5.19, p<0.01). However, forward transitions were hardly observed in conditions where either the visual or the auditory stimulus was turned off during the middle cycles. The transition was not significant in the reduced auditory adaptation condition (shift amount, 0.13±0.12 disk positions; paired t-test, t(7) = 1.089, p = 0.312). In the reduced visual adaptation condition, the transition was present (shift amount, 0.25±0.07 disk positions; paired t-test, t(7) = 3.654, p<0.01), but accounts for only 20% of the forward transition in the control condition (0.25 versus 1.22; paired t-test, t(7) = 5.41, p<0.001). These results indicate that when either visual or auditory stimuli were omitted for the intermediate cycles, the late cycles were judged essentially the same as the initial cycles–almost as if it was a fresh start of the stimulus. Therefore, adaptation to either of the modalities by themselves seems to play little role in producing the forward transition. Moreover, the similarity between the results of the reduced auditory or visual adaptation conditions makes it highly unlikely that the forward shift is merely due to a linear summation of these two adaptations. Thus, it is the simultaneous presentation of both modalities that seems to be critical, and the underlying mechanism should be something other than adaptation within a modality.

### Attentional distraction

The requirement that both audio and visual signals be simultaneously present implies that the forward transition effect is based on a continual process of crossmodal integration. Moreover, the state-transition analysis (Figure 3) showed that the percepts had a path dependency based on the observer's prior internal state. One possibility is that these internal states are mostly under bottom-up control and the transition pattern emerges automatically when observers are exposed to the stimuli. Another possibility is that these internal states reflect the attentional tracking of the simultaneity percept, and thus would be influenced by disruptions in attention. The disruption of forward shifts either by the omission of the visual or the auditory stimulus could be attributed to the fact that in those conditions, the position of current AV synchrony cannot be tracked with attention.

To test for the involvement of attention in the forward transition more directly, we examined whether distracting attention *away* from the stimuli could disrupt the forward shift. For this, we added a concurrent attentional task during the middle cycles. Observers were asked to count the number of ‘X’s in a letter stream. On half of the trials, the observers were required to perform this attention task, and on the other half, they were asked to ignore the letter stream.

**Figure 5:**
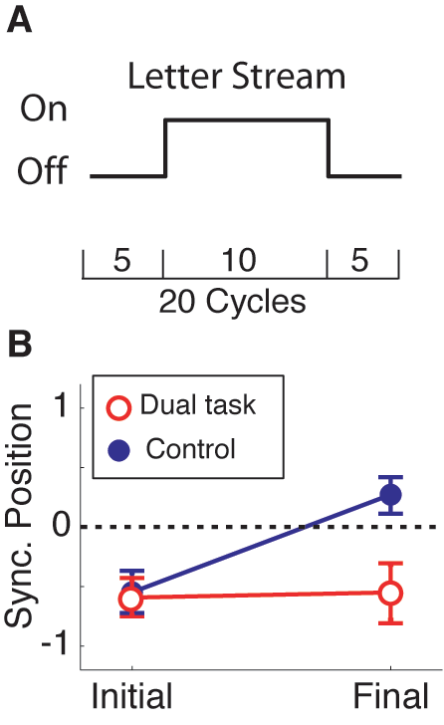
Attentional distraction experiment. A. A trial consisted of 20 cycles, and the letter stream was presented during the middle ten trials. The observers were asked to report the position of the position of perceptually synchronous disk for the initial five and the final five cycles. B. The perceptually synchronous positions for the initial and final cycles are plotted separately for the dual-task condition (open red circles) and for the single-task condition (solid blue circles). The error bars indicate one SEM (n = 6).

The results are shown in Figure 5B: When the observers performed the attentional task, the forward transition was completely abolished (paired t-test, t(5) = 0.474, p = 0.656). This is in contrast to the single-task condition in which a significant forward transition was obtained (paired t-test, t(5) = 5.772, p<0.01). The results indicate that AV synchrony needs to be tracked with attention for the forward transition to occur.

### Attentional modulation of AV synchrony

While the experiment above suggests some involvement of attention in the forward transition, the exact role it plays in the perception of AV synchrony remains unclear. In light of the known effects of attention on perception, two alternative hypotheses need to be considered. First, attention to a stimulus is known to speed up its processing and render its percept earlier than the percepts of unattended stimuli–a phenomenon known as *prior entry* [19], [28], [29]. A hypothesis derived on the basis of prior entry is that attention to a disk presented later than the physically synchronous disk should shift AV synchrony towards a later disk, whereas attention to a disk presented earlier than the synchronous disk should have little effect on the position of AV synchrony or possibly prevent the disk from perceived as synchronous with the auditory click.

An alternative possibility is that attention facilitates the binding between different modalities [30], that is, an attended disk is more preferentially bound to the auditory click. The binding hypothesis predicts that attention to a disk presented later than the physically synchronous disk should delay AV synchrony towards a later disk, whereas attention to a disk presented earlier than the synchronous disk should advance the position of AV synchrony to an earlier disk.

To examine these alternative hypotheses, we tested the effects of attention using three representative attention manipulation methods. First (pop-out experiment), we used a salient, pop-out stimulus: We presented a red disk at one of the eight locations and green disks at the other locations. This manipulation is expected to attract attention to the pop-out stimulus [31]. Second, we presented a cue (a small white disk lasting 40 ms) at one of the eight positions, just before the first cycle of a trial. This type of spatial cuing is known to grab attention [32]. Third, we manipulated the observers' overt attention, that is, we had the observers fixate directly on one of the disk locations [33]. In all three experiments, the relative positions of the attended target disk and the disk physically synchronous with the click were randomized across trials. Observers (n = 6) had to report the location of AV synchrony after five cycles of viewing.

**Figure 6:**
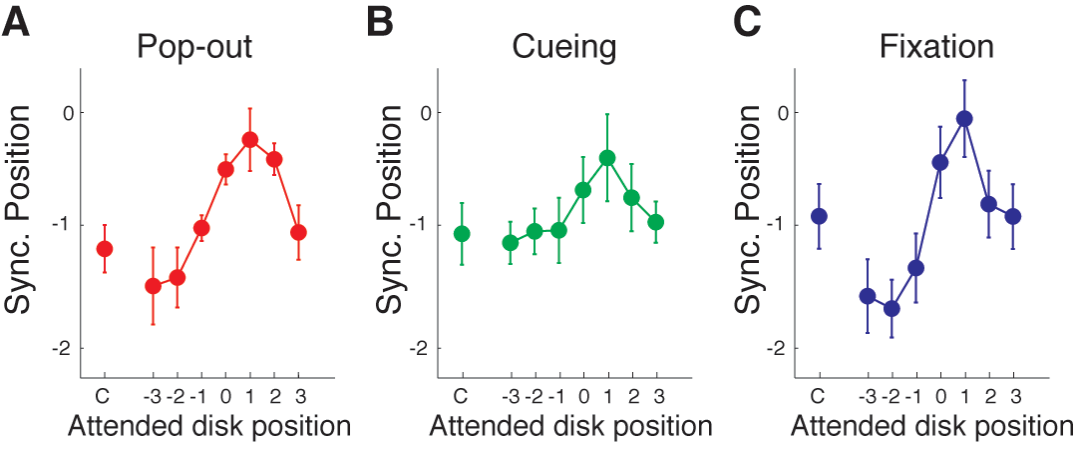
Results of the attentional manipulation experiments. (A–C) The mean PSP is plotted as a function of attended position for the pop-out (A), cueing (B) and fixation (C) experiments. The left most data points are the results of the control conditions in which no attentional manipulations were made. In all attentional manipulations, the position of a perceptually sycnhronous disk was most shifted in the forward direction and reached close to the veridical position when observers attended to the disk that occurred one disk later than the veridically synchronous disk. Error bars indicate one s.e.m. (n = 6).

The results are shown in Figure 6. In all three experiments, perceived AV synchrony was systematically biased towards the position of the attended disk (repeated measures ANOVAs: pop-out, F(5,35) = 5.00, p<0.01; cueing, F(5,35) = 27.60, p<0.001; fixation, F(5,35) = 18.97, p<0.001). When attention was directed to a disk presented earlier than the click, perceived AV synchrony was shifted to an earlier position. On the other hand, when attention was directed to a disk later than the click, perceived AV synchrony was shifted to a later position. In other words, AV synchrony was attracted towards the attended position. However, the effects of this attentional attraction were limited to the conditions in which attention was directed relatively close (<100 ms) to the physically synchronous disk.

One concern regarding the effect of attention is that observers reported the attended position when they were uncertain about the target position. Such a response bias might contribute to the attraction of AV synchrony towards attended position. One reason why we believe that the attraction effect is not simply due to the response bias is that the attraction was observed only in the cases where attention was directed to a disk near the veridically synchronous disk. If response bias was the only cause of the attraction effect, then attraction should have occurred regardless of the attended position relative to the veridically synchronous disk. However, this was not the case. In addition, in most conditions, the response was not peaked at the attended location, but a location between the veridical position and the attended location. This suggests that the attraction effect was a result of the interaction between low-level sensory signals and attention.

These results show that attention can both advance and delay the perceived timing of the attended visual stimulus relative to the timing of the sound. This argues against the idea that perceived AV synchrony was modulated by a simple facilitation of the processing speed for attended visual stimuli. Instead, the results support the hypothesis that attention facilitates the binding of a sound to the attended visual event regardless of its timing relative to that of the sounds.

The above results showing attraction of AV synchrony towards the locus of attention offers insights into the mechanisms underlying the forward transition. The forward shift can be accounted for by a combination of the attentional attraction effect and a tendency for observers' attention to be dragged forward in the direction of the visual motion. Visual motion would bias attention toward a slightly forward position from the currently attended, synchronous position [34], and thus one's percept of AV synchrony would more likely be pulled forward, and not backward, in the direction of motion. Once AV synchrony shifts to a new position, attention would also shift to that location with an additional bias in the forward direction. This recurring cycle of shifts in AV synchrony and attentional re-focusing can account for the dominance of the forward shift. The occasional backward transitions may occur when the current position of AV synchrony deviates from the near-veridical positions.

## Discussion

In this study, we reported a novel crossmodal illusion whereby the perception of AV synchrony fluctuates between different temporal positions. Perceptual alternations in multistable stimuli have been widely used in visual neurosciences to investigate the neural correlates of subjective perception under the presentation of a constant stimulus [21]. The multi-sensory display reported here can be used in a similar fashion to dissociate perceptual from stimulus-driven factors when one searches for the neural correlates of crossmodal temporal binding.

Our analyses revealed systematic transition patterns such as the cumulative forward shifts and the perceptual stability of each position of AV synchrony (>5 s; see Figure 2C). These patterns would not have been found if transitions were caused merely by random fluctuations in bottom-up signals. Rather, these systematic patterns indicate a dependency of subsequent perceptual state on the present perceptual/attentional state. The dominance of forward transitions over the entire period of a trial (Figure 2A and Figure 2B) can be taken as a signature of attentional involvement in the perceptual transitions of AV synchrony.

Perceptual binding across modalities seems to influence perceived timing: when visual and auditory stimuli are bound as a single event, their perceived timing is modulated to become simultaneous. In our experiments, the perceived timing of a disk relative to the timing of the click could both be advanced and delayed depending on where attention was directed. This finding defies a simple explanation based on facilitation of processing speed. Instead, it is better explained by the idea that attention facilitates the binding of the attended disk to the click and thus the point of perceptual simultaneity is attracted towards the attended stimulus. The importance of cross-modal binding in perceived timing has been suggested in earlier studies. A phenomenon relevant to the present study is temporal ventriloquism in which the perceived timing of a visual stimulus is typically attracted to that of the sound that the visual stimulus is bound with [35]–[36]. Another example is a spatial congruency effect: when a pair of AV stimuli come from the same spatial location, they are more likely to be judged as simultaneous than when they come from different locations [20]. These examples, among others, support the idea that when attention binds auditory and visual stimuli as originating from a common event, their relative timing is perceived as simultaneous.

At present, it is unclear what kind of mechanism underlies the attentional facilitation of audiovisual temporal matching. One possibility is that attention expands the temporal window of visual events [37], [38], as it does for perceived durations [39]. This might in turn increase the chance that the signals of attended visual stimuli temporally overlap with the auditory signals.

Inasmuch as this phenomenon involves spontaneous alternations between a number of mutually exclusive perceptual states, it resembles the class of multistable stimuli widely used in studies of visual perception [21], and might prove similarly useful for dissociating subjective report from physical stimulus input. However, it remains an open question whether the illusion reported here shares common mechanisms with classical rivalry stimuli such as binocular rivalry and moving plaids [40] as well as auditory bistable stimuli [41].

In summary, our present multistable illusion demonstrates that perception of simultaneity has a flexible nature, and is highly susceptible to attentional modulation. Moreover, our findings suggest that the perception of AV synchrony is not simply determined by the processing latency for each modality alone, but is constructed based upon perceptual binding of multisensory information as a common event. How exactly feature binding occurs across sensory modalities is a challenging problem, but the multistable stimuli reported in our present study may provide both an insight, and a paradigm for further studies into this issue.

## Materials and Methods

### Apparatus

The stimuli were generated on a G4 Macintosh computer and presented on a 22-inch CRT monitor (LaCie Blue Electron). The stimuli were viewed at a distance of 57 cm and head movements were restrained using a chinrest. The resolution of the monitor was 1024 by 768 pixels, and the refresh rate was 75 Hz. The auditory stimuli were presented through headphones (MDR-CD270, Sony Inc., Japan). The simultaneity of auditory and visual stimuli was assessed with a digital oscilloscope (Tektroniks TDS 210) and was accurate and stable over time.

### Continuous tracking

Ten observers (nine naïve observers and one of the authors, RK) participated. A white disk on a black background revolved about the fixation. The radius of the disks was 0.78 deg and the disks were presented at an eccentricity of 5.86 deg. The movement of the disk was a discrete apparent motion consisting of a sequential presentation of a disk at eight positions. Each disk was presented for 53.3 ms and there was a blank interval of 13.3 ms before the onset of the next disk (See, Figure 1a). The initial position of the disk was randomly chosen from the eight positions and the direction was randomly chosen from either clockwise or counter-clockwise. In each cycle, the onset of the 4th or 5th disk position from the initial disk position of a trial was accompanied by a click sound (approximately 70dB SPL). A new click sound was generated for each trial by assigning each sound frame a value randomly sampled from zero-centered Gaussian distribution with a sigma being half of the maximum intensity. The duration of the sound was 1 ms.

The factors defining a trial were counterbalanced within observer, resulting in a total number of 32 trials/observer ( = 8 [initial positions]×2 [sound locations]×2 [directions of rotation]). In a single trial, the stimulus sweep was repeated for 60 cycles. The task was to report the position of the synchronous disk continuously throughout the trial by pressing a key corresponding to the location. From the ten observers, we obtained the data of a total of 19200 cycles.

Unimodal adaptation experiments: The parameters for the stimuli were identical to the experiment above, but only 20 cycles were presented in a trial. The observers were asked to report the initial and final positions of the disk, which was perceived as synchronous with the click. For each observer, the initial and final estimates were calculated as the circular mean of the reported positions. In the reduced auditory adaptation condition, the click was not presented during the middle 10 cycles, and in the reduced visual adaptation condition, no visual stimulus but the fixation marker was shown during the middle 10 cycles. Eight observers including one of the authors (RK) participated. Each observer completed a total of 96 trials ( = 8 [initial positions]×2 [sound locations]×2 [directions of rotation]×3 [conditions]).

Attentional distraction experiment: For the dual task experiment, a letter stream was presented during the middle 10 cycles. The letters were presented in Helvetica font and their size was 1.0×1.2 on average. Each letter was presented for 120 ms. The observers were asked to report the number of occurrences of the letter ‘X’ in the stream. The number of Xs was varied between 3, 4 and 5. The mean performance was 92.2%. In the control conditions, the observers were encouraged to track the position of synchronous flash, while ignoring the letters. The order of these two conditions was counterbalanced across observers. Six observers including one of the authors (RK) participated in these experiments.

Attention manipulation experiments: Six naïve observers participated. The stimulus parameters for the disks and the sound were identical as the other experiments. For the pop-out condition, one of the disks was red, while other disks were all green. The luminance of the red and green were adjusted to near-isoluminant level. A control condition was intermixed in which all the disks were green. In the cue condition, the cue was a white disk with a diameter of 0.39 deg and was presented at the center of one of the disks 120 ms before the onset of the first disk in a trial. The disks were presented all in green (the same luminance was the pop-out experiment). In the control trials, no cue was presented. In the fixation experiment, the fixation marker was drawn directly on the one of the disk positions, and observers were required to fixate on the marker during a trial. In the control trials, the fixation marker remained in the center of the display.
